# Molecular Characterization of a Feline Panleukopenia Virus Strain Isolated from a Dog with Clinical Gastroenteritis in Spain

**DOI:** 10.3390/ani16182860

**Published:** 2026-09-11

**Authors:** Alberto Prieto, Silvia Valverde, Pablo Díaz, José Manuel Díaz-Cao, Gonzalo López-Lorenzo, David García-Dios, Renato Barrionuevo, Gonzalo Fernández

**Affiliations:** 1 Department of Animal Pathology (INVESAGA Group), Faculty of Veterinary Sciences, Campus Terra, Universidade de Santiago de Compostela, 27002 Lugo, Spain; alberto.prieto@usc.es (A.P.); silvia.valverde@rai.usc.es (S.V.); josemanueldiaz.cao@usc.es (J.M.D.-C.); gonzalo.lopezlorenzo@usc.es (G.L.-L.); d.garcia.dios@usc.es (D.G.-D.); gonzalo.fernandez@usc.es (G.F.); 2 IBADER—Instituto de Biodiversidade Agraria e Desenvolvemento Rural, 27002 Lugo, Spain; 3 Hospital Veterinario HA (VetPartners Spain), 27002 Lugo, Spain; 4 Institute for Research in Global Health and Sustainable Development (iTERRA), Campus Terra, Universidade de Santiago de Compostela, 27002 Lugo, Spain; 5 Unit of Reproduction and Obstetrics, Department of Animal Pathology, Faculty of Veterinary Sciences, Campus Terra, Universidade de Santiago de Compostela, 27002 Lugo, Spain; renato.barrionuevo@rai.usc.es

**Keywords:** feline panleukopenia virus, dogs, host range, VP2, sequencing

## Abstract

Canine parvovirosis is a fatal disease state traditionally caused by canine parvovirus (CPV), which evolved from feline panleukopenia virus (FPV). While FPV was historically restricted to felids, FPV-like strains were recently reported to be causing clinical disease in dogs. This study aims to characterize an FPV-like strain detected in an eight-week-old unvaccinated female dog in Spain exhibiting hemorrhagic diarrhea and severe leukopenia. After fecal DNA isolation, a 1745-base-pair fragment of the VP2 gene was sequenced for phylogenetic and molecular analysis. Our results revealed that this strain shared high homology with local feline FPV strains; the sequence was clustered near to other FPV strains detected in dogs elsewhere. Molecular analysis identified an isoleucine-to-threonine substitution at position 101 (I101T) of the VP2 protein. This mutation, common in contemporary FPV and CPV variants, has been described as a structural modification that optimizes interaction with the canine transferrin receptor, suggesting a potential role in canine host adaptation. In conclusion, this study represents the first report in Spain of FPV associated with clinical gastroenteritis in dogs. These findings underscore the necessity of continuous genomic surveillance of these highly evolving viruses for monitoring host-switching events.

## Simple Summary

Parvovirus infections are serious conditions that usually cause severe diarrhea and vomiting in cats or dogs. For a long time, it was believed that the cat strains of this virus could not cause disease in dogs, but in recent years some cases have been described outside of Spain. Our study reports the first case in Spain of a young dog with bloody diarrhea and low white blood cell counts infected with feline panleukopenia virus (FPV). In this strain, the genetic code of VP2—the main protein of the outer shell of FPV—was analyzed to detect alterations that could explain this cross-species transmission; thus, a specific mutation producing an amino acid change at position 101 was found. This mutation is found in almost all FPV strains detected in sick dogs worldwide, suggesting that it could be critical for this host switching. Therefore, our results support that FPV is increasingly becoming a more prevalent threat for dogs and highlights the need for constant monitoring of these highly evolving viruses.

## 1. Introduction

Canine parvovirosis is a serious disease characterized by acute hemorrhagic enteritis that is frequently fatal, especially in naïve or poorly immunized hosts [1]. The etiological agent of this disease is the canine parvovirus (CPV), a small, non-enveloped, single-stranded DNA virus first described in 1978 currently classified as *Protoparvovirus carnivoran1* species; since its emergence, the original strain of CPV has rapidly evolved into different genetic and antigenic variants distributed worldwide, which have completely replaced the ancestral one [2,3,4]. Different types of CPV are currently described based on the amino acid residue located at position 426 of the VP2 capsid protein: CPV-2a, CPV-2b, CPV-2c; in addition, two putative new types has also been proposed based on changes at position 297: new-CPV-2a and new-CPV-2b [5,6,7]. This VP2 protein is the major component of the viral capsid and is highly related to host affinity through the interaction with transferrin receptor (TfR) type 1 in host cells, acting as a molecular “key” that must perfectly fit into this receptor to initiate infection [8,9,10].

Despite being DNA viruses, the members of the *Protoparvovirus carnivoran1* species are characterized by a high substitution rate, estimated at approximately 1.09 × 10^−4^–1.79 × 10^−4^ nucleotide substitutions per site and year, a frequency remarkably similar to that of many RNA viruses [10]. Although there is a lack of direct evidence, it has been suggested that CPV evolved as a host variant of feline panleukopenia virus (FPV), possibly through transmission dynamics involving wild fauna [11]. In fact, this hypothesis is mainly supported by the detection of intermediate FPV-like variants in wild carnivore populations, the 98–99% nucleotide similarity between FPV and CPV and the *a priori* host range restriction of FPV [3,10]. In this regard, investigations analyzing the host range of both viruses revealed that all CPV variants have been identified in both healthy and clinical cats since they can infect different feline cells [12,13,14,15,16]. In contrast, FPV or FPV-like infections in dogs are occasional and limited to a small number of cases [12,17,18,19,20,21,22,23]. Historically, FPV was considered to have a highly restricted replication capacity in dogs, with experimental studies showing limited replication primarily in thymic and bone marrow tissues [16,24]. However, the recent identification of FPV-like viruses capable of efficient replication in canine cells and of causing systemic infection in dogs suggests that this host restriction may not be absolute [25]. Thus, current research is focused on the characterization of the VP2 sequence in CPV and FPV variants from both cats and dogs, in order to identify different mutations potentially associated with host range and pathogenicity [19,20,22,23]. These findings are essential for unraveling the evolutionary routes of these viruses and for assessing the role of FPV as a primary causative pathogen for dogs.

Related to this, current immunization strategies face significant challenges due to the rapid evolution of *Protoparvovirus carnivoran1*. In canine medicine, most commercial vaccines utilize modified live virus (MLV) formulations based on either the original CPV strain or the CPV-2b variant. While these formulations typically provide robust cross-protection against all circulating variants (2a, 2b, and 2c), reports of “vaccination failures” in adult dogs have become a subject of intense research [15,16]. The situation regarding feline populations and FPV circulation presents similar difficulties. Feline vaccines utilize the classic Cu-4 strain; however, a recent molecular survey revealed a significant genetic gap between this vaccine strain and contemporary field isolates, which could potentially reduce effectiveness [23]. Moreover, the frequent infection of cats with CPV variants adds complexity, raising concerns about whether FPV-based vaccines provide adequate cross-immunity in felines [15].

The epidemiological landscape is further influenced by the increasing density of pet populations and the close cohabitation of dogs and cats in urban environments and shelters. These settings act as “biological melting pots” where high viral loads and frequent interspecies contact favor the emergence of novel variants [16]. In cats, the occurrence of mixed infections—where an individual is simultaneously infected with FPV and CPV—provides opportunities for superinfection and increases the genetic heterogeneity of the viral quasispecies. This diversity acts as a reservoir of variants, some of which may possess the necessary traits to explore new host species or tissue tropisms [23].

Considering this background, the present study aims to characterize the complete VP2 sequence of an FPV-like strain detected in a dog with hemorrhagic diarrhea in Spain, analyzing the differences in its amino acid sequence compared with reference and local FPV and CPV strains.

## 2. Materials and Methods

### 2.1. Case Presentation

In June 2021, an eight-week-old female dog with no prior vaccination history was presented to a veterinary clinic in Lugo (NW Spain) due to severe gastrointestinal signs. The animal was lethargic and exhibited vomiting and yellowish, mucoid diarrhea. Physical examination revealed fever (40.4 °C), moderate dehydration (5–8%), and abdominal pain on palpation. Hematological analysis showed mild regenerative anemia (4.26 × 10^6^ erythrocytes/µL; hematocrit 31%), accompanied by leukopenia (2940 cells/µL) and neutropenia (763 cells/µL). Serum biochemistry revealed hypoalbuminemia, mildly decreased electrolyte concentrations (Na^+^ and Cl^−^), and a slight increase in blood urea nitrogen (BUN) and creatinine. As the diarrhea became hemorrhagic shortly after admission, a rapid immunochromatographic test for CPV, CCoV and *Giardia duodenalis* (PARVO-CORONA-GIARDIA, Petia Vet Health, O Porriño, Spain) was performed, yielding a positive result only for CPV. A fecal sample was subsequently submitted to the Infectious Diseases Unit of the Veterinary Faculty in Lugo for molecular confirmation.

Based on these findings, the patient was hospitalized and treatment was initiated; briefly, the dog was managed with intensive supportive care, including fluid therapy, antiemetic treatment, analgesia, antimicrobial therapy, and early enteral nutrition. Intravenous Ringer’s lactate solution (Hartmann Braun, Melsungen, Germany) was administered according to the degree of dehydration, with the volume prescribed during the first 24 h calculated to cover maintenance requirements, estimated fluid deficit, and ongoing gastrointestinal losses. Maropitant (1 mg/kg, SC, SID; Cerenia^®^ 10 mg/mL, Zoetis, Louvain-La-Neuve, Belgium) was used for control of vomiting, while omeprazole (1 mg/kg, IV, SID; Omeprazol Normon 40 mg EFG, Laboratorios Normon, Madrid, Spain) was administered for the prevention of gastroduodenal ulceration. Analgesia was provided with buprenorphine (0.01 mg/kg, IV, TID; Bupaq^®^ 0.3 mg/mL, VetViva Richter GmbH, Wels, Austria). In addition, metamizole (25 mg/kg, TID; Buscapina Compositum^®^ 500 mg/mL + 4 mg/mL, Boehringer Ingelheim Vetmedica GmbH, Ingelheim am Rhein, Germany) was administered for control of pyrexia, monitoring rectal temperature every two hours to avoid subsequent hypothermia until thermal stability was maintained for at least 12–24 h; in addition, this product also contains butylscopolamine, which is useful for the management of abdominal pain. Antimicrobial therapy consisted of amoxicillin–clavulanic acid (12.5–25 mg/kg, SC, SID; Synulox^®^ 140 mg/mL + 35 mg/mL, Zoetis, Louvain-La-Neuve, Belgium). Early enteral nutrition with a commercial gastrointestinal liquid diet was initiated 12 h after resolution of vomiting (Royal Canin GI High Energy Liquid, Royal Canin, Aimargues, France); feeding was initiated at 25% of the daily energy requirement and progressively increased by approximately 30% per day until full caloric intake was reached. Recombinant feline interferon omega (2.5 MU/kg, IV, SID for 3 consecutive days; Virbagen^®^ 10 MU, Virbac, Carros, France) was administered as an adjunctive treatment, as its use has been associated with reduced mortality and clinical severity in dogs naturally infected with canine parvovirus [26]. As hypoalbuminemia was not severe, frozen canine plasma transfusion was not considered. Clinical and laboratory monitoring was performed every 24 h and included measurement of body weight, blood glucose, total protein and albumin concentrations, complete blood count, and serum electrolytes, in order to stablish therapeutic adjustments. The dog showed a gradual clinical improvement throughout treatment and, after seven days of hospitalization with clinical and laboratory monitoring, was considered clinically stable and returned home in good condition.

### 2.2. PCR Analysis and Sequencing

Firstly, DNA was isolated and purified using a commercial kit specifically optimized to remove stool-derived PCR inhibitors, which combines mechanical and chemical lysis with silica membrane-based purification (HigherPurity™ Stool DNA Isolation Kit, Canvax, Valladolid, Spain); the extraction was performed using 200 mg of fecal sample, following the manufacturer’s instructions at every step. Subsequently, a conventional PCR assay targeting a 1745 bp fragment of the VP2 gene of FPV/CPV was performed using primers P1 (5′-ATGAGTGATGGAGCAGTTC-3′) and VPR (5′-TTCTAGGTGCTAGTTGAG-3′) as previously described [27]. PCR reactions were performed in a final volume of 25 μL containing 2.5 U of NZYTaq II DNA Polymerase, 1×x Reaction Buffer, 1× Optimizer Solution, 2 mM of MgCl_2_, 0.2 mM of dNTPs (all reagents supplied by NZYTech Lda., Lisbon, Portugal), 0.5 μM of each primer (Invitrogen Custom Primers, Thermo Fisher Scientific, Carlsbad, CA, USA), and 5 μL of template DNA. Amplification was carried out in a Bio-Rad T100 thermocycler (Bio-Rad Laboratories Inc., Hercules, CA, USA) under the following thermal set-up: initial hot start at 95 °C for 60 s; 10 touch-down cycles (95 °C for 20 s, 55 °C for 30 s, and 68 °C for 60 s), decreasing the annealing temperature by 0.5 °C per cycle; 25 cycles (95 °C for 20 s, 50 °C for 30 s, and 68 °C for 60 s); and a final extension at 68 °C for 3 min. Two positive controls (previously confirmed FPV and CPV samples) and a negative control (HyPure Molecular Biology Grade Water, HyClone Laboratories, South Logan, UT, USA) were included. PCR products were visualized by electrophoresis on a 1% agarose gel in 1× TAE buffer stained with RedSafe™ (iNtRON Biotechnology Inc., Boston, MA, USA). Finally, PCR products were purified and sequenced by Sanger sequencing at an external service provider (StabVida, Lisbon, Portugal). The chromatograms obtained from both forward and reverse strands were assembled and manually proofread using ChromasPro 2.1.4 (Technelysium, Brisbane, Australia). The consensus sequence obtained was initially compared with those deposited in the GenBank NCBI database using the BLAST tool (https://blast.ncbi.nlm.nih.gov/Blast.cgi?PROGRAM=blastn&BLAST_SPEC=GeoBlast&PAGE_TYPE=BlastSearch accessed on 7 September 2026) available at the National Center for Biotechnology Information (NCBI). 

### 2.3. Phylogenetic Analysis

Prior to phylogenetic tree construction, the obtained sequence was aligned with selected sequences using the ClustalW multiple alignment algorithm implemented in MEGA X version 10.2.2 [28]; briefly, previously reported FPV sequences detected in dogs worldwide, as well as both reference and representative regional FPV and CPV sequences previously obtained from the study area (PZ279922-PZ279924 and PZ279926-PZ279932), were included in the analysis. In addition, a nucleotide homology matrix with all the employed sequences was generated using BioEdit 7.2.5 [29]. A mink enteritis virus sequence (GenBank accession no. M23999) was included in the alignment as an outgroup to infer ancestral relationships. Subsequently, the best fitting nucleotide substitution model was selected by using the Akaike Information Criterion (AIC) implemented in jModelTest v.2.1.10 [30,31]. A phylogenetic tree was constructed using MrBayes 3.2.7 [32], applying a Bayesian approach with Markov Chain Monte Carlo sampling (20,000,000 generations with sampling every 1000 steps). The resulting tree was visualized and edited using FigTree 1.4.3. Finally, the nucleotide alignment was also translated into amino acid sequences to identify amino acid changes between the VP2 sequence obtained in this study and previously reported FPV sequences detected in dogs elsewhere.

## 3. Results

The sample from the clinical case (CP50FPV) tested positive by conventional PCR, confirming the presence of viral DNA. After comparing our sequence with those deposited in GenBank, the highest nucleotide homology (99.89%) was observed in an FPV strain identified in a domestic cat from Spain (GenBank accession no. PQ436979). The novel sequence obtained in the present study was deposited in GenBank under accession number PZ279925.

**Figure 1:**
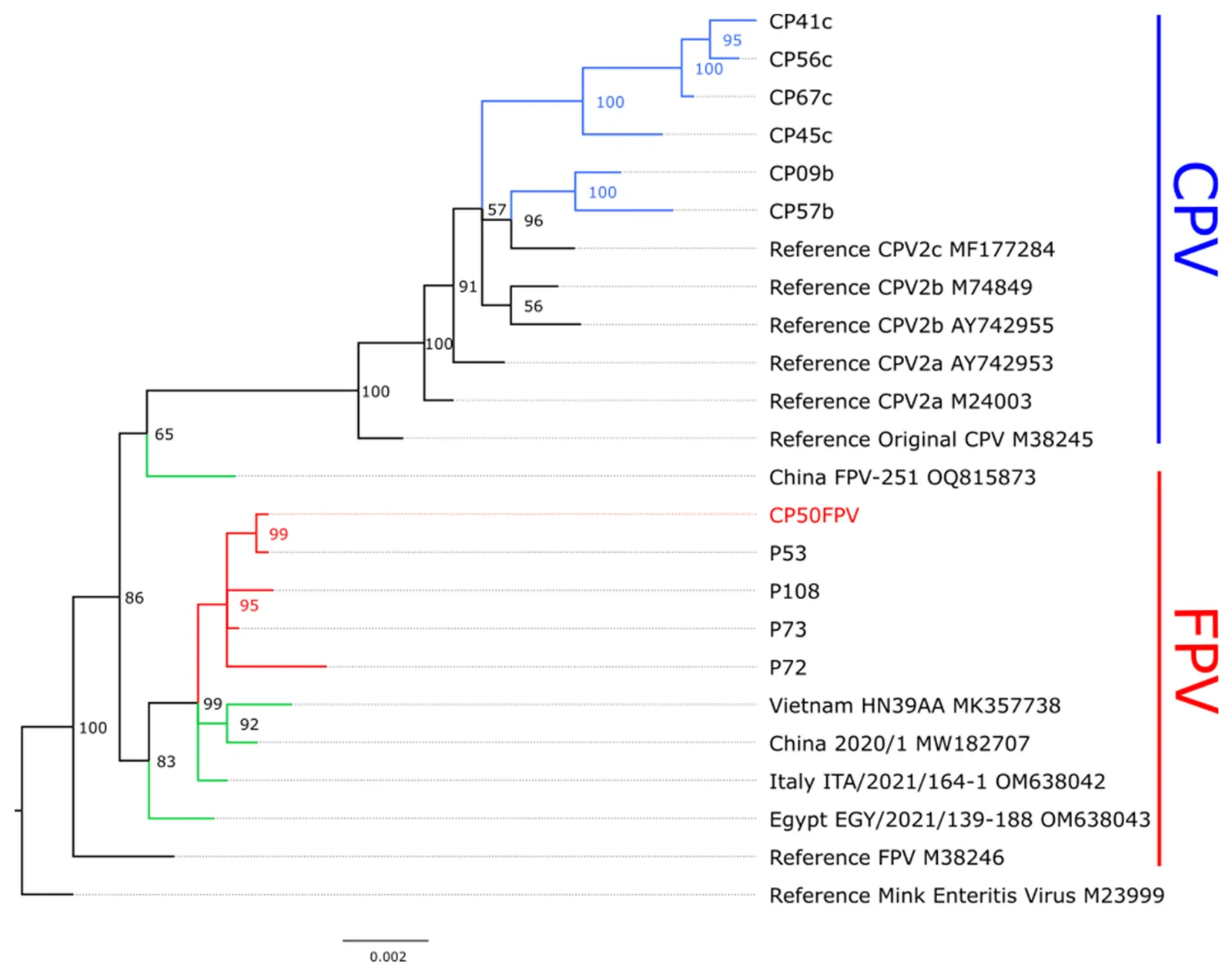
Bayesian phylogenetic tree showing the relation among FPV and CPV reference sequences and those obtained from the study area. Green clade: FPV sequences obtained from dogs elsewhere; red clade: FPV sequences from cats in the study area including the novel PFV strain (CP50FPV) detected in a dog the present study; blue clade: CPV sequences from dogs in the study area.

The phylogenetic tree is shown in Figure 1. According to the Akaike Information Criterion (AIC), the best-fitting nucleotide substitution model was the HKY model with a proportion of invariable sites. As can be observed, the separation between FPV and CPV sequences is clearly defined, although one previously described FPV sequence of Chinese origin (OQ815873) is positioned between both clades. Regarding the sequence obtained in the present study, it was clearly included within a well-defined clade with other FPV strains obtained from cats from the study region, suggesting that this strain likely originated from the local feline population. Similarly, this group of sequences also clusters with most FPV sequences previously described in dogs elsewhere. These observations are further supported by the sequence analysis results. Thus, the CP50FPV isolate showed nucleotide identity values ranging from 99.6% to 100% when compared to FPV strains from the same study region, whereas homology was lower (98.1–98.6%) compared with CPV strains from the same area. Similarly, the nucleotide identity with other FPV strains previously reported in dogs elsewhere ranged from 99.4% to 99.8%. 

Table 1 summarizes the amino acid changes observed among the analyzed sequences. As shown, our CP50FPV sequence exhibits a substitution of isoleucine to threonine at position 101 (I101T) relative to the FPV reference strain M38246, while the rest of the amino acid sequence is identical. This mutation I101T is also present in all FPV strains previously reported in dogs from other parts of the world; however, these strains also show at least an additional glycine-to-alanine substitution at position 568 (G568A) which is not present in the CP50FPV sequence.

## 4. Discussion

The biological boundary that traditionally excluded FPV from infecting dogs has become increasingly blurred in recent years. Historically, FPV was considered a virus showing restricted tropism because its replication in dogs was mainly limited to primary lymphoid tissues without resulting in significant viral shedding via the fecal route [10,22,24]. However, the emergence of CPV in the 1970s—derived from FPV or very closely related ancestors in wild carnivores—demonstrated the potential of this viral family to colonize new niches through minimal genetic changes [2,10,11]. At present, a number of recent studies from various countries, including Vietnam, Thailand, China, Japan, Pakistan, Egypt, and Italy, have identified FPV strains in dogs associated with clinical gastroenteritis, suggesting an active evolutionary process in which FPV is acquiring the capacity to overcome the species barrier [12,17,18,19,20,21,22,23]. Despite the limitation of being based on a single case, to the best of the authors’ knowledge, the present study represents the first report of an FPV strain detected in a dog with clinical signs compatible with parvovirosis in Spain, and the second such report in Europe. Nevertheless, it should be noted that, although the sample tested negative for CCoV and *G. duodenalis*, other potential enteric pathogens, including *Salmonella* spp. and *Campylobacter* spp., were not specifically investigated. Thus, although the marked leukopenia and neutropenia observed in the dog were more consistent with the systemic effects commonly associated with parvoviral infection, the contribution of other infectious agents to the clinical presentation cannot be completely excluded.

Most studies indicate that the *VP2* gene plays a key/major role in host range specificity since it encodes the major protein of the viral capsid and, consequently, defines the external architecture of the virus [34]. In addition, this protein also has a decisive role in interacting with the host’s transferrin receptor type 1 (TfR), a critical step for cellular entry and for determining the range of susceptible species [35,36]. Regarding this, available data suggests that a complete genomic overhaul is not required for FPV adaptation to dogs, but rather point mutations in strategic amino acid residues improving affinity for this canine TfR receptor [22,23].

The available literature has proposed a number of molecular changes associated with FPV host specificity, including the substitution of isoleucine for threonine at position 101 (I101T) in VP2, which has been proposed as a critical event [37]. While prototypical or older FPV strains retained isoleucine, contemporary variants have almost universally adopted threonine at this site, such as the strain CP50FPV identified in this study [19,20,21,22,23,38]. This mutation is not simply random genetic drift, since residue 101 lies at the core of the receptor-binding region and overlaps with an epitope recognized by neutralizing antibodies. Bioinformatic analyses have shown that the substitution to threonine at this position facilitates a polar interaction with residue Asp99, modifying the electrostatic charge and topography of a canyon-like structure on the capsid surface; thus, it was suggested that this structural adjustment optimize interaction with TfR, enabling the virus to more efficiently explore new hosts as canine species [19]. Nevertheless, the specific role of this VP2 conformational change in host adaptation and interspecies transmission remains to be experimentally validated.

The frequency of the I101T mutation is remarkably high in current isolates both in strains obtained from cats and in the canine-associated cases reported to date [19,20,21,22,23]. For example, all FPV strains detected in dogs from Vietnam presented this variant [19]. It is worth noting that this same mutation (Thr101) was consistently identified in CPV-2a, 2b and 2c variants when compared to original CPV strains, reinforcing the idea that threonine at position 101 is nearly indispensable for viral persistence in the canine population [19,22]. The ubiquity of this mutation in modern FPV populations—even in cats, as it was also found among the feline FPV sequences in this study—suggests that FPV already possesses one of the molecular changes necessary for host switching.

However, the I101T mutation appears to be only one piece of the puzzle. Recent research has identified other substitutions that could act as powerful determinants of host tropism. A particularly impactful finding has been the identification of the A300P mutation in FPV strains isolated directly from cats [23]. Thus, FPV isolates harboring this specific mutation have demonstrated an unprecedented ability to replicate stably and at high titers in canine cell lines such as MDCK and A72, differently from conventional FPV strains. Moreover, pathogenicity studies have confirmed that these variants not only replicate in vitro but are also capable of infecting dogs via the oral route, causing intestinal lesions, mesenteric lymphadenitis, and viral shedding in feces, and thereby mimicking the clinical behavior of CPV. However, no amino acid substitution was observed at position 300 of the VP2 protein in either the strain analyzed in the present study or the strains reported in previous studies, as shown in Table 1 [19,20,21,22,23]. Similar to this, another mutation of interest in this evolutionary process is the lysine-to-asparagine substitution (K93N) in residue 93, considered one of the key changes that enabled the original FPV to evolve into CPV and recently detected in FPV strains isolated from dogs with diarrhea in Thailand [20]. Nevertheless, most FPV strains detected in dogs worldwide, including the one found in the present study, lack this specific amino acid substitution, suggesting that it is unlikely that K93N mutation plays a critical role in adaptation to canine hosts [19,21,22,23].

Regarding the emergence of these FPV canine-adapted variants, it has been proposed that feline susceptibility to both FPV and CPV can facilitate natural coinfections in this species, creating a permissive environment for genomic recombination [15,16]. Although the sequence of the present study was not characterized as a recombinant strain, it should be noted that this phenomenon may generate intermediate FPV-like variants with chimeric capsid properties facilitating their entry and efficient replication in dog cells [17]. Consequently, cats can represent a crucial reservoir for the emergence of recombinant protoparvoviruses with the potential to overcome species barriers. A thorough analysis of available molecular epidemiological data, including the results of the present investigation, indicates that FPV is not a static agent; in this regard, the phylogenetic inference demonstrates that current circulating FPV strains isolated from cats are genetically distant from those classical FPV strains on which current vaccines are based, agreeing with previous reports [23]. This genetic gap, combined with a high selection pressure and close contact between dogs and cats, represents an ideal scenario for the emergence of FPV variants with expanded host tropism. Thus, detection of FPV in dogs is not a diagnostic error but a biological reality supported by the identification of key VP2 mutations. In relation to the present study, the described case involves an animal from a rural area where interaction between domestic dogs and cats is frequent, thereby facilitating exposure to species-specific viruses. In addition, FPV outbreaks commonly occur every year among cats in these kinds of rural areas in Spain, particularly during the kittening season, making the presence of the virus in the environment highly likely. This fact may increase dogs’ exposure to the virus, thereby increasing the likelihood of interspecies transmission; however, further epidemiological studies in the area would be valuable to characterize FPV circulation in local cat and dog populations and to better assess the potential risk of this phenomenon. In other hand, it is noteworthy that the patient was an eight-week-old puppy, an age that represents a critical window of susceptibility for parvovirus infection. This period coincides with the decline of maternally derived antibodies (MDAs), creating an ‘immunity gap’ that leaves the individual vulnerable to field strains. This susceptibility was further compounded by the fact that the animal had not yet received its primary vaccination series against core diseases, including canine parvovirosis. Consequently, it is plausible that the inherent host-related factors of this patient predisposed the individual to the development of overt clinical disease following FPV infection. Nevertheless, continuous surveillance of these clinical cases is imperative to determine whether this host-switching phenomenon occurs with a greater frequency than previously anticipated.

## 5. Conclusions

This report constitutes the first documented case of an FPV-like strain causing disease in dogs in Spain. Although this is a preliminary study based on a single case and with several limitations, the presence of the I101T mutation in canine-associated FPV strains, including the strain identified in the present study, appears to be a common feature among FPV strains detected in dogs worldwide and may therefore be associated with adaptation to canine hosts. In this context, monitoring these molecular changes, particularly in the VP2 gene, together with functional studies aimed at determining their biological significance, is warranted to better assess the potential establishment of these “host-switching” strains.

## Figures and Tables

**Table 1:** VP2 amino acid substitutions at relevant positions in the FPV strain obtained in this study regarding reference sequences and other FPV strains detected in dogs elsewhere.

Sequence Name	Amino Acid Position												Country	Accession Number	Reference
	80	93	101	103	232	300	323	375	411	562	564	568			
Reference FPV	K	K	I	V	V	A	D	D	E	V	N	G		M38246	[33]
CP50FPV	-	-	T	-	-	-	-	-	-	-	-	-	Spain	PZ279925	This study
HN39AA	-	-	T	-	-	-	-	-	-	-	-	A	Vietnam	MK357738	[19]
FPV-251	-	-	T	-	I	P	-	-	A	L	-	A	China	OQ815873	[23]
ITA/2021/164-1	-	-	T	-	-	-	-	-	-	-	-	A	Italy	OM638042	[22]
EGY/2021/139-188	-	-	T	-	-	-	-	-	-	-	-	A	Egypt	OM638043	[22]
FPV-VT2020	-	N	T	-	-	-	-	-	-	-	-	A	Thailand	MN270937	[20]
2020/1	-	-	T	-	-	-	-	-	-	-	-	A	China	MW182707	[21]
Reference CPV	R	N	I	A	I	-	N	N	-	-	S	-		M38245	[33]

## Data Availability

The original sequence (CP50FPV) presented in the study is openly available in the GenBank Database at https://www.ncbi.nlm.nih.gov/genbank/ (accessed on 7 September 2026) under accession number PZ279925. In addition, the remaining FPV and CPV sequences analyzed in this study—obtained from the same geographical area—were deposited under accession numbers PZ279922–PZ279924 and PZ279926–PZ279932.
